# Culturally Adapting Mindfulness Based Cognitive Therapy for the Chronic Pain Related Needs of Older Black Adults

**DOI:** 10.1093/geroni/igaf122.859

**Published:** 2025-12-31

**Authors:** Tony Pham

**Affiliations:** Massachusetts General Hospital, Boston, Massachusetts, United States

## Abstract

Up to 78% of older Black individuals experience chronic musculoskeletal pain. Among older Black individuals, chronic pain co-occurs with depression and early cognitive decline, and these co-morbidities worsen physical function. Addressing chronic pain and its co-morbidities among older Black adults is a public health priority. We utilized the Community Engagement (CE) Studio Toolkit to examine (1) barriers and facilitators to participation in Mindfulness-Based Cognitive Therapy (MBCT) and (2) perceptions of MBCT skills. We facilitated facilitated CE Studio focus groups among older Black individuals with chronic pain and depression (n = 31 across 6 groups). We thematically analyzed the CE Studio focus group transcripts using a hybrid deductive–inductive approach. Based on our analyses, five themes emerged about how MBCT should be culturally adapted for the chronic pain, depression, and early cognitive decline needs of older Black individuals. CE Studio members suggested that MBCT (1) address relevant socio-cultural issues; (2) include coping strategies already accepted by the Black community; (3) reducing the access barriers to MBCT by offering it for a less expensive stipend and in local group settings; (4) focus on providers who are sensitive to the older black community’s cultural, spiritual, and sociological needs (e.g., by expanding the definition of MBCT instructors to include lay instructors; and (5) abbreviating MBCT’s duration from 2- to 1-hour sessions. MBCT, culturally tailored to address the unique needs of older Black adults in the community, may be an effective and efficient solution to improve physical, emotional, and cognitive outcomes among this underserved population.

